# Public sector implementation strategies to approach the judicialization of health care: a systematic review protocol

**DOI:** 10.1186/s13643-022-02043-x

**Published:** 2022-08-10

**Authors:** Sueli Miyuki Yamauti, Jorge Otávio Maia Barreto, Silvio Barberato-Filho, Fernanda Lacerda da Silva Machado, Luciane Cruz Lopes

**Affiliations:** 1 Graduate Program in Pharmaceutical Science, University of Sorocaba, Rodovia Raposo Tavares, Km 92,5 —, Sorocaba, SP Brazil; 2 Oswaldo Cruz Foundation, Fiocruz, Brasília, DF Brazil; 3 Federal University of Rio de Janeiro, UFRJ, Macaé, Brazil

**Keywords:** Judicialization of health care, Organization and administration, Public health, Lawsuits and litigation, Justice administration system, Delivery of health care, Health systems

## Abstract

**Background:**

The judicialization of health care can be understood as a societal response to pressing healthcare needs, which probably are not being adequately addressed by the current healthcare system. This phenomenon increases the strain on service resources, both in the health sector and in the judiciary system as well, demanding an institutional reorganization to manage judicial actions. It is believed that each institution has developed strategies for coping with the problem in isolation and with little public disclosure. The purpose of this review will be to identify institutional strategies implemented and to investigate their effects in approaching the judicialization of health care.

**Methods:**

Electronic searches will be conducted up to December 2021 on 11 databases, supplemented with gray literature and references lists of secondary studies. The descriptor “judicialization of health” will be the basis for conducting the main research. Studies describing any strategy implemented by public institutions to approach the judicialization of health care will be included. Results related to the quality of services provided by the implemented strategy reported in the studies and those that report accessibility, usability, and potential adverse effects or harms caused by the implemented strategy will be investigated. In addition, it will be explored if there have been changes related to the value or characteristics of health litigation. Two reviewers will independently screen all citations, abstract data, and full-text papers. The risk of bias in each study will be appraised using a tool suggested by Cochrane Effective Practice and Care Organization Group (EPOC). Subsequently, the reviewers will also extract the data of interest and classify the findings of these studies according to their performance at the institutional level. The results obtained will be described as a narrative synthesis.

**Discussion:**

This review may provide evidence on the effects of the strategies implemented to approach the judicialization of health care. It will potentially benefit health care and legal professionals, decision-makers, and researchers by identifying the types and characteristics of strategies that have the potential to improve service delivery in the future.

**Systematic review registration:**

PROSPERO CRD42020160608

**Supplementary Information:**

The online version contains supplementary material available at 10.1186/s13643-022-02043-x.

## Background

Health is considered a human right [1], supported by the World Health Organization [2], and well-defined, in full and unrestricted terms, in the national constitutions of at least 61 countries [3]. However, these legal rights do not always translate into actual access to healthcare technologies. The term healthcare technology comprises the knowledge, materials, and services that can be applied by healthcare professionals and communities to cure or ameliorate diseases or injuries to the health of individuals, or the population in general [4], and include medications, equipment, hospital infrastructure, medical services, and other related categories [5].

Restrictions upon universal access to healthcare technology, whether due to budgetary issues, lack of inventory forecasting, or limitations in coverage of insurance system, reflect a deficient healthcare system [6]. In these cases, judicialization is recognized as the societal response to pressing healthcare needs, which probably are not being adequately addressed by the current health services [7]. Judicialization has been increasingly considered an alternative way to gain access to healthcare technologies [8].

### Description of the issue

The judicialization of health care, in part, reflects a social and participatory evolution of the citizen [9], but it can be a barrier to the successful realization of the collective right to health care [10]. It is a multifaceted phenomenon that is growing more intensely in Latin America and the Caribbean [8, 11]. In these regions, lawsuits are filed against a health authority due to gaps in health policy and/or failures in its implementation [11], with a very high probability, almost certainty, which the ruling will favor the claimant [12].

The accelerated and cumulative growth of healthcare lawsuits has alarmed both healthcare managers and jurists, urging debates [6] concerning the legitimacy of judicial interference in health care, the costs of judicialization, and the limits on public expenditure to not undermine the implementation of current public health policies [6, 13].

The increased demand for services and financial resources in the healthcare area as well as in the judiciary system [12, 14–16] forced an institutional reorganization, reallocating scarce resources which otherwise would be dedicated to the delivery of health care [13].

### Description of the strategy

Public health and legal institutions have implemented a wide range of strategies to approach the judicialization of health care.

This protocol adopted the term “strategy” to define any action or service, experience or practice, or support for decision-making, which has been used by public institutions to approach the judicialization of health care [17].

The systematic review will investigate the institutional strategies that have been implemented to avoid or minimize the negative effects of judicialization on the public health system. These strategies were implemented to resolve disputes extrajudicially (before filing a lawsuit) or during a judicial process or to organize the institutional services (internal organization of work processes) to prevent litigation [17]. Table 1 shows the classification of implemented strategies and their components that will be adopted in the systematic review.

**Table 1:** Classification of implemented strategies and their components according to institutional level

Institutional level	Classification of strategies	Components
Service organization	Health Technical Information Services	•Specialized multiprofessional teams •Evidence-based rapid response services •Information and communication systems •Educational meetings and training •Elaboration and updating of protocols and therapeutic guidelines of clinical practice •Health technology assessment, selection, and incorporation •Standardized and organized documents and procedures •Interinstitutional agreements
	Information and communication systems	•Use of computers, Internet, and software •Logistic systems with inventory control •Information on lawsuits, plaintiffs, patients, and service provision •Registration of technical information •Online communication •Document upload •Technical and management reports
	Service qualification	•Educational meetings and training •Standardized and organized documents and procedures •Information and communication systems •Elaboration of statements, care protocols, and therapeutic guidelines •Implementation of specialized services •Audit and monitoring of services
	Healthcare provision	•Multiprofessional teams •Specialized pharmacies •Centralized control and monitoring of lawsuits, plaintiff information, and products in stock •Standardized and organized documents and procedures •Financial resources to finance individual health care or to deposit a sum of money into the plaintiff’s bank accounts to pay for the claimed health technology •Information and communication systems
Nonjudicial approach	Administrative proceedings	•Information and communication systems •Specialized pharmacies •Standardized and organized documents and procedures •Criteria for the provision of services •Citizen orientation and referral to the health unit responsible for the requested care •Health Technical Information Services •Interinstitutional agreements
	Alternative dispute resolutions	•Information and communication systems •Use of mediation or conciliation •Standardized and organized documents and procedures •Criteria for the provision of services •Health Technical Information Services • Interinstitutional agreements
Judicial approach	Defense of the health authority	•Attorney or health workers specializing in health or law respectively •Standardized and organized documents and procedures •Information and communication systems •Health Technical Information Services •Interinstitutional agreements

### How the strategies might work?

The strategies can be implemented at various stages of access to health care, from the search to the effective provision of services to citizens.

They can be divided according to their performance at the institutional level:

1. Organization of services: strategies implemented in public institutions aiming to organize work processes following public policies and government programs. They may include the creation of support services.
2. Nonjudicial or extrajudicial approach: strategies that favor dialogue between claimant and public institutions without filing a lawsuit.
3. Judicial approach: strategies used at any point during the judicial process, including the enforcement of court decisions. They may provide expert health technical support to judges and courts or to defend the health authority [17].

Strategies focused on the organization of services and extrajudicial approaches aim to avoid lawsuits by facilitating the delivery of healthcare services. They may include the provision of technologies not covered by the healthcare system, conditioned to the availability of financial resources and the fulfillment of medical and legal requirements [17].

**Fig. 1:**
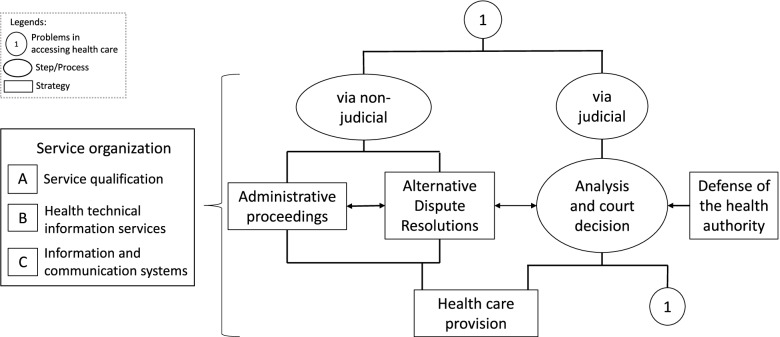
Diagram of a logical model using some implementation strategies to approach the judicialization of health care

Strategies implemented in judicial sector aim to support the judge’s decision-making through the analysis of judicial processes or the availability of technical reports based on scientific evidence in the health area. Such strategies are expected to reduce attempts of corruption and the impacts of the judicialization of health care on institutional resources [17] (Fig. 1).

### Why it is important to do this review?

It is believed that each institution has developed strategies for coping with the problem in isolation. Information about these experiences regarding the planning, implementation, or evaluation may occasionally be described in institutional administrative documents and, in some cases, local legislation [7, 13, 17]. Therefore, mapping studies or successful experiences that can guide or be replicated in other healthcare services or institutions is challenging.

Previous publications on the subject revealed that many studies have only characterized the lawsuits (including the profile of litigants, physicians and lawyers, and the healthcare technologies involved in lawsuits) [18–21]; however, there exist few studies describing or analyzing the strategies adopted by public institutions to manage these lawsuits [16].

Given this scenario, the purpose of the review will be to scrutinize the literature to identify and investigate the effects of the implemented strategies used by the public sector to approach the judicialization of health care.

## Methods

### Protocol registration and reporting

This systematic review protocol is being reported in accordance with the reporting guidance provided by the Preferred Reporting Items for Systematic Reviews and Meta-Analyses Protocols (PRISMA-P) criteria (Guidelines checklist) [22]. The review protocol has been registered within the International Prospective Register of Systematic Reviews (PROSPERO) on November 30, 2019, and was last updated in December 2021 (registration number CRD42020160608) [23].

### Eligibility criteria

#### Types of studies

Non-randomized trials, before-and-after controlled studies, interrupted time series, and repeated measures studies will be included. The documents will be included if they are in English, Spanish, and Portuguese, without restrictions of date or country. There will be no restriction on publication status.

#### ECLIPSE framework

The ECLIPSE framework is a set of structured questions used as an alternative aid to help in the search for information on health and social care management [24].

##### Expectation

We look for strategies or good practices implemented in public institutions, both in the health and judicial sectors, aiming to approach the judicialization of health care.

##### Client group

They are people who have not been able to access public health services and who are willing to file a lawsuit to claim their rights.

##### Location

It is across the entire public health and judicial sectors involving public healthcare issues.

##### Impact

Reduction or avoidance of health litigation, improvement, or expansion in access to health services, promotion of rational use of health technologies and institutional resources, reduction of expenses with health litigation, and improvement in communication between professionals.

##### Professionals

These include health professionals, mainly physicians, pharmacists, nutritionists, and nurses; legal professionals, attorneys, defenders, public lawyers, judges; and managers of public institutions.

##### Service

They are public institutions that deal with the judicialization of health care. For example, in the healthcare sector, this includes the Ministry of Health, state, municipal, or regional health departments, hospital care, outpatient services, and pharmaceutical services. In the judicial sector, this includes the Court of Justice, Public Prosecution Service, Public Defender Service, State Attorney’s Office, and City Attorney’s Office.

#### Types of outcome measures

##### ***Use of the implemented strategy***

Any parameter reported in studies to measure the effectiveness of the implemented strategy is as follows:

- Rate of use of technical reports and notes prepared by the implemented strategy, by the legal department, or by court of law
- Adherence rate of citizens, magistrates, defenders, or promoters to the implemented strategy
- Adherence rate of doctors, magistrates, lawyers, defenders, or attorneys to institutional guidelines and recommendations
- Attendance rate of people served versus not attended

##### ***Quality of service delivery***

Any measure that assessed an improvement in the provision services is as follows:

- Waiting time rate for user service, for the acquisition of health technologies, or for the provision of health care
- Resolution rate
- Patient satisfaction rate with the health care provided

##### ***Measures related to the institution and the professional involved***

- Acceptability rate or worker satisfaction with the implemented strategy
- Assessment of worker productivity after strategy implementation
- Difference between the costs of judicialization of health care before and after strategy implementation
- Difference between institutional costs before and after strategy implementation

##### ***Equity***

Any reports that addressed issues about inequalities caused in the populations served by the strategy implemented, according to the classification of the PROGRESS-Plus tool [25, 26], for example,  the implementation of a single waiting list for organ donors for transplants.

##### ***Measures related to lawsuits***

Any changes detected in the characteristics of the lawsuits after the strategy is implemented:

- Changes in the pattern or type of lawsuits and changes in the profile of claimant
- Mean difference between requests granted and not granted
- Mean difference between lawsuits and administrative proceedings
- Reduction in the number of lawsuits filed
- Proportion of successful lawsuits to rejected lawsuits
- Rate of extrajudicial resolutions

##### ***Adverse effects or harms***

Unintended consequences caused by the implemented strategy that may result in adverse effects or harms are as follows:

- Increased time spent by providers to perform administrative tasks
- Failures in verbal or nonverbal communication
- Privacy and information disclosure issues
- Failure or delay in the delivery of health technology
- Loss or misuse of health technologies

#### Exclusion criteria

Studies that merely describe or characterize lawsuits in the healthcare area (for example, type of lawsuit, characteristics of patron, number of lawsuits) will be excluded. In addition, studies reporting the motives of claimants or discussing, from a conceptual or theoretical point of view, possible and not implemented strategies to approach the judicialization of health care will not be included.

Finally, strategies aimed at promoting the judicialization of health (for example, legal activism, right to death, abortion or reproduction, compulsory hospitalization) will also be excluded.

### Information sources

Electronic searches will be tested and performed by two reviewers in the following electronic databases:

1. Electronic databases: Portal de Periódicos da Coordenação de Aperfeiçoamento de Pessoal de Nível Superior do Brasil (Periódicos CAPES) (which includes the following databases: OneFile, Scopus, Social Sciences Citation Index, Expanded Sciences Citation Index (Web of Science), Directory of Open Access Journals, Sociological Abstracts, MEDLINE/PubMed, Scientific Electronic Library Online (SciELO), SciELO Brazil, JSTOR Archival Journals, NDLTD Union Catalog, SAGE Journals and Publications, Science Direct Journals and Books, Elsevier, Oxford Journals, Materials Science & Engineering Database, Cambridge Journals, Dialnet, Embase), Cumulative Index to Nursing and Allied Health Literature, and Virtual Health Library Regional Portal-BVS (which included LILACS)
2. Electronic database specialized in public health: the Cochrane Library, Health System Evidence, and Epistemonikos
3. Gray literature: websites of government agencies, health ministry or department, justice ministry or department, non-indexed journals, public institutions that were cited in the documents found, and other sources of gray literature that may be considered relevant for the systematic review

### Search strategy

A search with the keyword “judicialization of health” (in Portuguese, judicialização da saúde), according to the descriptors described by the Health Sciences Descriptors (DeCS) of Brazil [27], will be performed in PubMed to identify a predefined sample of studies. The main words of the descriptor will be used separately and in combination with Boolean operators (OR and AND), in Portuguese, English, and Spanish: [(judicialização OR judicialización OR judicialisation OR judicialization) AND (saúde OR health OR salud) (Additional file 1).

Other related descriptors, and their synonyms, will also be tested. The final version of the search strategy will be adapted to be used in other information sources in accordance with the specificities of each selected database.

In the databases The Cochrane Library, Health System Evidence, and Epistemonikos, we will conduct the searches using the term judicialization of health and generic terms such as legal, judicial, and litigation. This adaptation is justified by the fact that judicialization of health care is not a widely used descriptor, being only recently incorporated into the DeCS [27] (since 2017), and it is scarcely used in the relevant literature outside of Latin America.

Searches in electronic databases will be conducted until December 2021, and the same searches will be re-executed just before the final analysis.

### Study records

#### Screening of studies

Prior to the data collection process, calibration exercises will be conducted to ensure consensus among reviewers. After a consensus is achieved, each reviewer will receive a reference manager (EndNote X7) containing all the exported searches without duplicates.

Reviewers will work in pairs and independently to select titles and abstracts using the eligibility criteria. When there are disagreements between the pairs of reviewers, a third reviewer will be consulted.

A new calibration exercise will be conducted before the full-text study distribution. The full texts of eligible studies will be grouped and distributed among reviewers. The same pair of reviewers will evaluate these documents and identify studies for inclusion. Also, they will record the reasons for the exclusion of the ineligible studies.

Two independent reviewers will scrutinize the reference list of secondary studies to identify potentially eligible articles. They will also perform a hand search for eligible articles in unindexed journals that publish on the subject. In the case of disagreements between the reviewers, a third reviewer will be consulted.

The systematic review will begin after the publication of this protocol and is expected to be completed in December 2022.

#### Data extraction and management

Two reviewers will independently extract the data from the full texts according to a pre-piloted and standardized data-extraction template with fill-in instructions, using the Excel 2016 software.

The extracted data will include the following information:

1. Specific details about the characteristics of the study (author, year, title, type of publication, design)
2. Specific details about the strategy implemented (name of the strategy, year, and location, objectives, actors involved, limitations, and possible harms caused by the strategy, barriers, and facilitators to implement the strategy)
3. Population and setting characteristics
4. Study duration
5. Outcomes measured
6. Funding sources and competing interests

If an implemented strategy is reported in several publications, only data from the study considered to be the most complete will be extracted.

In cases when a study has an outcome reported in different measurement units, or if the results have many variables or items, the variable that was considered the most important by its authors will be selected. In the absence of this criterion, the reviewers of the systematic review will choose, by consensus, the most appropriate data to be collected for this study.

If there are disagreements between two reviewers about any information to be extracted from the studies collected, a third reviewer will be consulted. If needed, reviewers will contact the lead author of the study to clarify any doubts.

#### Risk of bias in individual studies

A criteria-based tool to analyze the risk of bias for systematic reviews suggested by the Cochrane Effective Practice and Organization of Care Group (EPOC) will be applied for studies conducted with a separated control group, such as non-randomized trials, before-and-after controlled studies, interrupted time-series, and repeated measures studies [28].

The findings will be grouped by similar practices (characteristics and objectives) and analyzed together. Subsequently, the reviewers will assess whether the biases found are sufficiently relevant to interfere with the interpretation of the results.

#### Synthesis of results

The systematic review will be reported according to the Preferred Reporting Items for Systematic reviews and Meta-Analyses (PRISMA 2020) statement [29] and synthesis without meta-analysis (SWiM) guidelines [30].

The strategies implemented to approach the judicialization of health care identified in this study will be grouped, analyzed, and discussed according to their similarities, and the respective clusters will be coded according to Table 1.

Considering the diversity of strategies and outcomes measured, we anticipate that the available data will not be sufficient to group the estimate and perform a meta-analysis. Therefore, the findings will be reported as a narrative synthesis [31].

The narrative synthesis will be elaborated considering three aspects:

1. How the strategy worked and how it affected the judicialization of health care
2. What are the effects of the strategy implemented on issues involving health inequities. We will use the PROGRESS-Plus framework (place of residence, race/ethnicity, occupation, gender, religion, education, socioeconomic status, social capital, and complemented with age, disability, and sexual orientation) [25, 26] to investigate aspects related to the inequity in health
3. What are the barriers and facilitators interfering with the implementation of the selected strategies. We will use the SUPPORT tools (SUPport POlicy relevant Reviews and Trials) to guide analyses of the barriers and facilitators that affect the implementation of a strategy and its implications for health, the health system, users, policymakers, and professionals involved [32].

#### Meta-bias

A comprehensive search strategy associated with an extensive list of electronic databases and gray literature sources will be employed to obtain a greater number of publications to be scrutinized. If sufficient data is found, funnel plots and Egger’s test [33] will be used to assess the risk of publication bias.

#### Confidence in cumulative evidence

The Grading of Recommendations, Assessment, Development and Evaluations (GRADE) tool [34] will be used to assess the strength of the evidence and to issue the degree of recommendation of the strategies. For each outcome found, the set of domains, methodological limitations, indirectness, imprecision, inconsistency, and the likelihood of publication bias, will be classified according to the quality of evidence: high (the true effect lies close to that of the estimate of the effect), moderate (the true effect is likely to be close to the estimate of the effect, but there is a possibility that it is substantially different), low (the true effect may be substantially different from the estimate of the effect), and very low (the true effect is likely to be substantially different from the estimate of effect) [34].

Two reviewers independently will assess the certainty of the evidence and resolve discrepancies through a third reviewer.

## Discussion

The implementation of an institutional strategy to approach judicialization should be carefully monitored using a robust methodology capable of informing the decision-maker about the real benefits of the intervention.

The evaluation and monitoring of any strategy are needed since it can point out barriers and facilitators for its implementation, indicating areas for improvement according to the local context. Consequently, the results will be improved, and the application of resources can be optimized, with potential positive impacts on the quality and access of services by citizens.

It is expected that the systematic review will provide crucial information on the effectiveness of strategies implemented in public institutions to approach the judicialization of health care and insights regarding how these strategies can be replicated or adapted to similar contexts.

Policymakers and stakeholders will be able to use the systematic review to support the planning and implementation of more efficient and comprehensive healthcare policies, such as cost-effective programs and services that can potentially reduce inequities in access to healthcare technologies.

In addition, this study is expected to identify and report gaps in scientific knowledge about the judicialization of health in public health systems, including the management, financing, and delivery arrangements, providing data that can be useful to guide further research.

Any changes made to this protocol when conducting the study will be described in PROSPERO and the final manuscript.

### Strengths and limitations of this study

- Studies that investigate the strategies implemented in the public sector to approach the judicialization of health care are scarce and difficult to access. Therefore, it is useful to provide an overview of these strategies so that they can be disseminated to a wider audience.
- The main challenge of the systematic review will be able to adequately group and interpret the results of different types of studies and implemented strategies.
- The elaboration of a narrative synthesis and an evidence gap map will better describe how public services have organized to approach the judicialization of health care.

## Supplementary Information

**Additional file 1.** Examples of search strategy. Guidelines checklist. PRISMA-P (preferred reporting items for systematic review and meta-analysis protocols) 2015 checklist: recommended items to address in a systematic review protocol.

## Data Availability

Not applicable. Data sharing is not applicable to this article as no datasets were generated or analyzed during the current study.

## Abbreviations

DeCS: Health Sciences Descriptors
ECLIPSE: Expectation, Client group, Location, Impact, Professionals, SErvice
EPOC: Cochrane Effective Practice and Organization of Care Group
GRADE: Grading of Recommendations, Assessment, Development and Evaluations
Periódicos CAPES: Portal de Periódicos da Coordenação de Aperfeiçoamento de Pessoal de Nível Superior do Brasil
PRISMA-P: Preferred Reporting Items for Systematic Review and Meta-Analysis Protocols PRISMA 2020 Preferred Reporting Items for Systematic reviews and Meta-Analyses
PROGRESS-Plus Framework: Place of residence, race/ethnicity, occupation, gender, religion, education, socio-economic status, social capital, and complemented with age, disability, and sexual orientation
PROSPERO: Prospective Register of Systematic Reviews
SciELO: Scientific Electronic Library Online
SUPPORT: SUPporting POlicy relevant Reviews and Trials
SWiM: Synthesis without meta-analysis
