# Supplementary material for: Public sector implementation strategies to approach the judicialization of health care: a systematic review protocol
Source: Syst Rev. 2022 Aug 10;11:164. doi: 10.1186/s13643-022-02043-x (PMC9364593; doi:10.1186/s13643-022-02043-x)
Supplement: Supplementary file 1 — Additional file 1. Examples of search strategy. Guidelines checklist. PRISMA-P (preferred reporting items for systematic review and meta-analysis protocols) 2015 checklist: recommended items to address in a systematic review protocol. [file 13643_2022_2043_MOESM1_ESM.pdf]

Additional file 1. Examples of search strategy

1. Advanced searches in the Periódicos CAPES:

Boolean operators	Search terms
AND	(judicialização OR judicialization OR judicialisation OR judicialización)
	(saúde OR health OR salud)

2. MEDLINE (via PubMed):

Nº	Search terms
#10	((((judicialização) OR judicialización) OR judicialization) OR judicialisation) AND (((saúde) OR salud) OR health)
#9	(((OR judicialização) OR judicialización) OR judicialization) OR judicialisation
#8	judicialização
#7	judicialización
#6	judicialization
#5	judicialisation
#4	((saúde) OR salud) OR health
#3	saúde
#2	salud
#1	health
